# Uneven Meibomian Gland Dropout in Patients with Meibomian Gland Dysfunction and *Demodex* Infestation

**DOI:** 10.3390/jcm11175085

**Published:** 2022-08-30

**Authors:** Xinxin Yu, Yana Fu, Hengli Lian, Dandan Wang, Zuhui Zhang, Qi Dai

**Affiliations:** 1 School of Optometry and Ophthalmology, The Eye Hospital of Wenzhou Medical University, Wenzhou 325027, China; 2 Eye Center, The Second Affiliated Hospital, Zhejiang University School of Medicine, Hangzhou 310009, China; 3 The First Affiliated Hospital of Soochow University, Suzhou 215006, China; 4 College of Mathematical Medicine, Zhejiang Normal University, Jinhua 321004, China

**Keywords:** meibomian gland dysfunction, *Demodex* infestation, uneven atrophy score

## Abstract

The purpose of this study was to compare the differences between uneven meibomian gland (MG) atrophy with and without *Demodex* infestation based on the index of uneven atrophy score (UAS). In this retrospective cohort study, 158 subjects were recruited, including 66 subjects in the *Demodex*-positive MGD group, 49 subjects in the *Demodex*-negative MGD group, and 43 subjects as normal control. No significant difference was verified in OSDI, TMH, TBUT, CFS, lid margin score, and meibograde (all *p* > 0.05) between the *Demodex*-positive MGD group and the *Demodex*-negative MGD group. The UAS index of the upper eyelid or both eyelids was significantly higher in the *Demodex*-positive group in comparison with the normal control group and *Demodex*-negative group and the difference was statistically significant between the three groups. The UAS was significantly positive correlation with OSDI (r = 0.209, *p* < 0.05), lid margin score (r = 0.287, *p* < 0.001), and meibograde (r = 0.356, *p* < 0.001), which has a significant negative correlation with TBUT (r = −0.248, *p* < 0.05). Thus, *Demodex* infestation can cause uneven MG atrophy and we propose a novel index of UAS, which is used to evaluate uneven atrophy of MGs and as a morphological index of Demodex infestation.

## 1. Introduction

*Demodex* mites are the most common photosensitive microscopic ectoparasite inhabiting the human skin [1,2]. The incidence rate of *Demodex* infestation increases with age, being observed at nearly 100% among people over 70 years [3]. There are two distinct species that affect human skin: *Demodex* folliculorum and *Demodex* brevis [4,5]. *Demodex* folliculorum resides in the eyelash follicles, and *Demodex* brevis are capable of burrowing deep into sebaceous glands and meibomian gland (MG) [6,7]. Once the facial skin is infected, it is likely to spread and enrich in the eyes, leading to ocular demodicosis. Mites can cause not only blepharitis and chalazion, but also marginal corneal infiltration, phlyctenule-like lesions, superficial corneal opacity, meibomian gland dysfunction (MGD), and so on [8,9,10].

**Figure 1:**
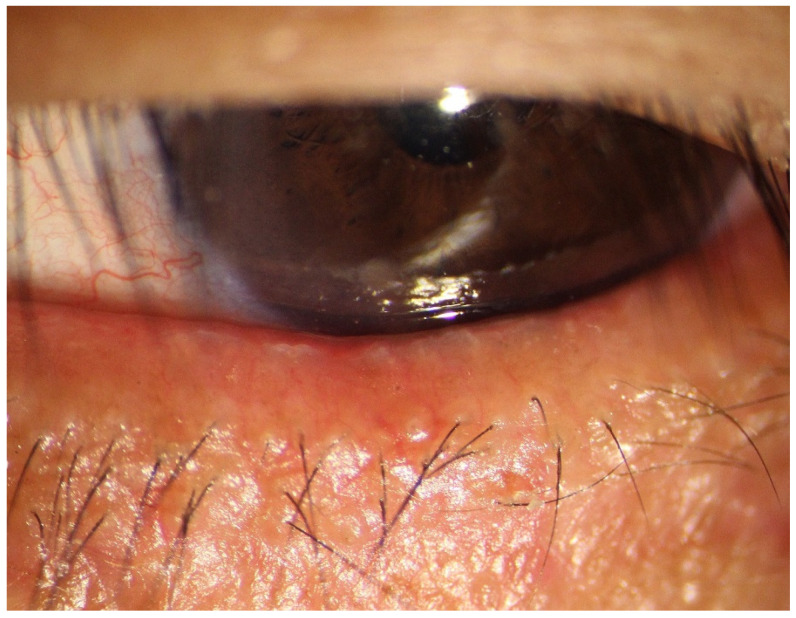
**The slit-lamp photo of the *Demodex* infested eyelid.** The photo showed marked diffuse hyperemia of lid margin, lid margin telangiectasia, and abnormal eyelashes with cylindrical dandruff.

In recent years, many investigators have reported that ocular demodicosis has a significant correlation with ocular discomfort, lid margin abnormality, corneal epithelial barrier disruption, and MGD [11]. *Demodex* mite infestation can give rise to inflammation of lid margin (Figure 1), leading to properties alteration of MGs secretion, and mechanically blocking the sebaceous ducts [12,13,14]. Pan et al. [15] indicated that *Demodex* positive group showed significantly increased scores of ocular surface disease index (OSDI), ocular redness score, and MG dropout. However, Rabensteiner [9] and Huo [16] et al. discovered that there had been no significant difference in MG dropout with or without ocular *Demodex* infestation. Therefore, more evidence will be needed to testify correlation between the *Demodex* mite infestation and MG dropout.

MG dropout is considered a sign of MGD. Previous studies have mainly focused on the decrease of meibomian gland density [17], gland distortion, gland tortuosity [18], gland ghost, and so on. Clinically, we observe that MG atrophy is more uneven in patients with *Demodex* mite infestation. Yin et al. [19] found that uneven meibomian gland dropouts existed in patients with MGD, in which the nasal and temporal dropouts were higher than the middle dropout. However, there is no precise and accurate definition of uneven MG atrophy at present. Therefore, in our study, the parameter of uneven atrophy score (UAS) was introduced to evaluate the uneven MG atrophy. The purpose of this study is to compare the differences in uneven MG atrophy with and without *Demodex* infestation based on the index of UAS.

## 2. Materials and Methods

### 2.1. Subjects

We conducted a retrospective cohort study at the Affiliated Eye Hospital of Wenzhou Medical University from June 2019 to September 2020. This study was approved by the Research Ethics Committee of the Eye Hospital, Wenzhou Medical University (approval number: 2020-209-K-191). All procedures adhered to the tenets of the Declaration of Helsinki. Informed consent to publish was obtained from all participants before inclusion in the study. This study is registered on http://www.clinicaltrials.gov/ (accessed on 30 June 2020) (NCT04451122). All subjects were recruited by systematic random sampling from the dry eye outpatient and divided into three groups (normal control group, *Demodex*-negative MGD group, and *Demodex*-positive MGD group). Only the right eye was included in this study. Exclusion criteria included a history of ocular trauma or surgery; acute ocular surface inflammation; wearing contact lenses within the past two weeks; other ocular or systemic diseases affecting MG function. The total sample size of the three groups is mainly based on PASS 15 software. In a single factor ANOVA study, sample sizes of 32, 37, and 43 are obtained from the 3 groups whose means are to be compared. The total sample of 112 subjects achieves 92% power to detect a difference of at least 0.65 using the Dunnett (With Control) multiple comparison test at a 0.0500 significance level. The common standard deviation within a group is assumed to be 0.57.

### 2.2. Diagnosis of MGD and Demodex Infestation

MGD was diagnosed based on the presence of ocular surface-related symptoms, lid margin abnormality score (irregular shape; vascular engorgement; bulge, ester cap, or plug in MG orifices), poor meibum quality, and a poor meibum expressibility score [20].

*Demodex* infestation was diagnosed based on Chinese diagnostic criteria of *Demodex* blepharitis [21]. *Demodex* infestation positive: three eyelashes were removed from each eyelid under a slit-lamp microscope. Next, *Demodex* mites were detected and counted by a professional technician under a light microscope. Subjects were diagnosed with *Demodex* infestation, in which more than three mites were found on any eyelid in any stage, such as mites, whether dead or alive, larvae, and eggs. The *Demodex*-negative MGD group was a MGD group alone without *Demodex* infestation or suspected *Demodex* infestation.

### 2.3. Ocular Surface Examination

The parameters of ocular surface assessments were as follows: OSDI questionnaire, tear meniscus height (TMH), tear break-up time (TBUT), corneal fluorescein staining (CFS), lid margin abnormality score, and meibography. The subjects filled out the OSDI questionnaire, in which the questionnaire gives a range of 0 (no symptoms) to 100 (severe symptoms), and then TBUT and CFS were measured under fluorescein staining. TBUT was measured three times, and the mean value was recorded. Lid margin abnormalities were graded using the Baylor grading scheme from 0 to 4, including: anterior or posterior displacement of the mucocutaneous junction, vascular engorgement or lid margin neovascularization, plugged meibomian gland orifices, and irregularity of the lid margin [22]. The Keratograph 5M (K5M; Oculus, Wetzlar, Germany) was used to perform the meibography scans of the upper and lower eyelids and to acquire meibograde and TMH.

### 2.4. Uneven Atrophy Score (UAS)

A senior and experienced physician manually annotated the area of the upper or lower tarsal plate and MGs by VIA software (2.0.3., http://www.robots.ox.ac.uk/∼vgg/software/via/ (accessed on 25 October 2018)) and calculated the ratio of computer-aided MG loss. Next, we converted the ratio of MG loss into meibograde using a validated meibograde grading scheme. A validated meibograde grading scheme, with a four-point scale from 0–3: grade 0: area of MG loss between 0–25%; grade 1: area of MG loss between 26–50%; grade 2: area of MG loss between 51–75%; and grade 3: area of MG loss 76–100% [23]. The tarsus area was evenly divided into five equal parts according to the transverse diameter of the tarsus by a computer program based on Numerical Python (V0.0.2, https://github.com/riga/numphy (accessed on 3 May 2018)), and the MG atrophy was calculated in every part of the upper or lower eyelid. We also evaluated the repeatability between two measurements by the same observer at different time points in order to avoid subjective errors.

**Figure 2:**
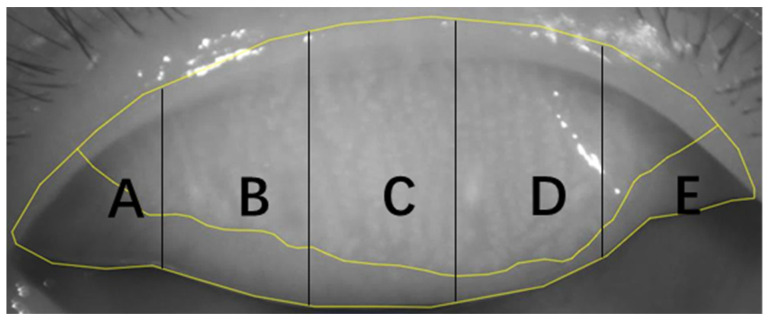
**The computational method of UAS**. UAS = |meibograde A−meibograde B|+|meibograde B−meibograde C|+|meibograde C−meibograde D|+|meibograde D−meibograde E|. The ABCDE represent different areas in tarsal plate.

We introduced the concept of uneven atrophy score (UAS) based on the total variation. The total variation for a function with one variable is used to measure the magnitude of its oscillations, standing for its uneven properties. The UAS is obtained by summing the absolute values of the difference between MG loss grades in different regions in turn (Figure 2):

$$\mathrm{UAS}=\left|meibograde\;A-meibograde\;B\right|+\left|meibograde\;B-meibograde\;C\right|+\left|meibograde\;C-meibograde\;D\right|+\left|meibograde\;D-meibograde\;E\right|\;\left|x\right|=\{\begin{array}{c} -x,\;x<0\\ x,\;x\geq 0 \end{array}$$

**Figure 3:**
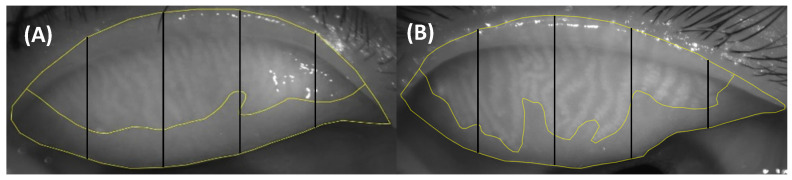
**The different UAS between the two meibography images of similar meibograde (meibograde = 1).** (**A**) showed the UAS (UAS = 0) of the upper eyelid in the *Demodex*-negative MGD group, and (**B**) showed the UAS (UAS = 3) of the upper eyelid in the *Demodex*-positive MGD group.

Generally, the larger the UAS, the more uneven the MG atrophy. Figure 3 showed UAS between the two meibographs of similar meibograde.

### 2.5. Repeatability of UAS

To measure the repeatability of UAS, 19 subjects were randomly selected using a random number table. The meibography images of these patients were used for reproducibility analysis. To calculate intra-observer variation, the area of the tarsal plate was circled two times by the same operator at two different time points. Next, MG loss grade for each part of the eyelid was calculated two times. Finally, we obtained the UAS by the same operator at two different time points.

### 2.6. Statistical Analysis

Statistical analysis was performed using SPSS 26.0 statistical software. Values are expressed as the mean ± standard deviation (SD) or (range) or median (interquartile range [IQR]). The normality of all datasets was tested by using the Kolmogorov–Smirnov test. One-way Analysis of Variance or Kruskal–Wallis H test was used to compare differences between the 3 groups. The generalized estimating equation was used to adjust the age difference. A *p*-value < 0.05 was considered significant. The correlations between UAS index and MG function parameters (i.e., OSDI, TBUT, and lid margin score) and meibograde were determined using Spearman’s correlation analysis. To evaluate the repeatability between two measurements at different time points, Bland–Altman analysis was obtained using MedCalc version 19.0.4 (MedCalc, Ostend, Belgium). The 95% limits of agreement (LoA) were calculated as the mean difference ± 1.96 SD.

## 3. Results

### 3.1. Basic Characteristics and Ocular Surface Parameters

In this cross-sectional study, 158 subjects were recruited from the dry eye outpatient, Eye Hospital, Wenzhou Medical University, Zhejiang, China. They were divided into three groups: 66 subjects were in the *Demodex*-positive MGD group (20 males and 46 females), 49 subjects were diagnosed with *Demodex*-negative MGD group (19 males and 30 females), and the other 43 subjects were in the normal control group (18 males and 25 females). The demographic features and ocular surface parameters were presented in Table 1. The median age of the subjects in *Demodex*- positive MGD group was 38.5 (28.0, 55.5) years and in the *Demodex*-negative MGD group was 40.0 (30.0, 48.0), which were significantly older than subjects in the normal control group (25.0 (22.0, 32.0)). There were significant differences in OSDI, TBUT, lid margin score, and meibograde between the normal control group and the *Demodex*-negative MGD group or between the normal control group and the *Demodex*-positive MGD group (all *p* < 0.05). No significant difference was verified in OSDI (*p* = 0.120), TMH (*p* = 0.569), TBUT (*p* = 1.000), CFS (*p* = 0.424), lid margin score (*p* = 0.442), and meibograde (*p* = 1.000) between *Demodex*-positive MGD group and *Demodex*-negative MGD group. The TMH did not have a statistically significant difference between the three groups (all *p* > 0.05).

### 3.2. Uneven Atrophy Score between Three Groups

Table 2 showed the UAS of MGs and meibograde in the normal control group, *Demodex*-negative MGD group, and *Demodex*-positive MGD group. The UAS of the upper eyelid was statistically significant between the three groups. Compared with the other two groups, subjects with *Demodex* mite infestation were significantly higher (all *p* < 0.05). Since age was statistically significant among the three groups, a generalized estimation equation was used to adjust the age using for comparing differences between the three groups. The UAS of the upper eyelid was still statistically significant between the normal control group and the *Demodex*-positive group or between the *Demodex*-negative group and the *Demodex*-positive group (all *p* < 0.05). There was a statistically significant difference in lower eyelid between the *Demodex*-negative group and the *Demodex*-positive group (*p* = 0.020). The normal control group and *Demodex*-positive group had no statistically significant difference after adjusted for age (*p* = 0.213) in the lower eyelid. The UAS of both eyelids was the same as the upper eyelid (all *p* < 0.05). The UAS index was significantly higher in the *Demodex*-positive group in comparison with the normal control group and *Demodex*-negative group. In the meantime, we also compared the meibograde in the normal control group, *Demodex*-negative group, and *Demodex*-positive group. The meibograde of the upper eyelid had a statistically significant difference between the normal control group and the *Demodex*-negative group or between the normal control group and the *Demodex*-positive group (all *p* < 1.000 adjusted for age). There was no statistically significant difference in meibograde of the upper eyelid between the *Demodex*-negative group and the *Demodex*-positive group (*p* = 0.547). The meibograde of the lower eyelid or both eyelids were the same as the upper eyelid. In addition, Table 3 showed the number or ratio of UAS in the normal control group, *Demodex*-negative MGD group and *Demodex*-positive MGD group. When the UAS score was between 0 and 3, the proportion of the number in the *Demodex*-positive MGD group was significantly lower than that in normal control group and *Demodex*-negative MGD group. However, when the UAS score was between 7 and 9, the proportion of the number in the *Demodex*-positive MGD group was significantly higher than that in normal control group and *Demodex*-negative MGD group. No one in in normal control group and *Demodex*-negative MGD group had a UAS score between 10 and 12.

### 3.3. Correlations between Uneven Atrophy Score and Ocular Surface Parameters

**Figure 4:**
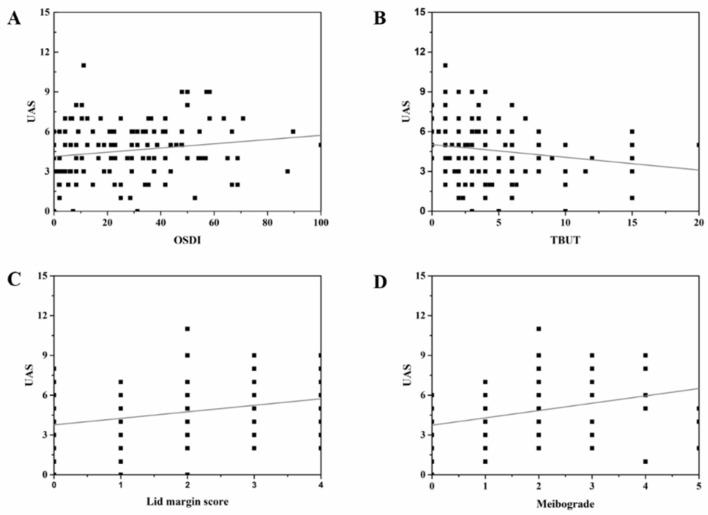
**Correlations between UAS and OSDI, TBUT, Meibograde, and Lid margin score.** (**A**) showed the correlation between UAS and OSDI, (**B**) showed the correlation between UAS and TBUT, (**C**) showed the correlation between UAS and Meibograde, and (**D**) showed the correlation between UAS and Lid margin score.

To understand the relationship between UAS and ocular surface parameters, Spearman correlation analysis was performed. The UAS of both eyelids had significantly positive correlation with OSDI (r = 0.209, *p* < 0.05), lid margin score (r = 0.287, *p* < 0.001), and meibograde (r = 0.356, *p* < 0.001), which has significant negative correlation with TBUT (r = −0.248, *p* < 0.05). However, there were no significant correlations between UAS of both eyelids and CFS (r = −0.008) or TMH (r = 0.064). The results of the upper eyelid were consistent with both eyelids. The results were shown in Table 4 and Figure 4.

### 3.4. The Intra-Observer Repeatability of UAS

**Figure 5:**
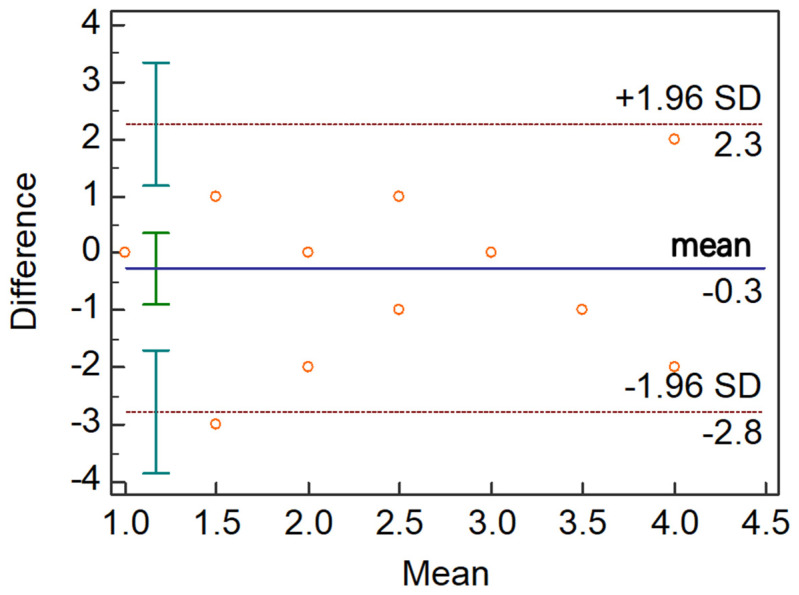
**The Bland–Altman plots of intra-observer repeatability differences for UAS.** The 95% LoA for UAS showed high agreement between the two measurements at different time points.

Figure 5 showed the Bland–Altman plots of the difference between the two measurements by the same observer. The 95% LoA for UAS showed high agreement between the two measurements at different time points. Overall, 94.7% of the points were within the 95% LoA, which indicated that the intra-observer repeatability was good.

## 4. Discussion

Ocular *Demodex* infestation causes abnormalities in the ocular surface, lash follicles, and MGs, triggers an inflammatory response in the host, and could cause a delayed hypersensitivity reaction, particularly in patients with rosacea [24,25,26,27,28]. In addition, MGD is a very common MG disease and *Demodex* might cause damage to the meibomian gland, leading to MGD.

In our study, a comparison between the *Demodex*-positive MGD group, *Demodex*-negative MGD group, and normal control group showed that the normal control group was significantly lower than the *Demodex*-negative MGD group and the *Demodex*-positive MGD group in symptom, lid margin abnormalities, and MG dropout, and was significantly higher in TBUT. No significant difference was found between the *Demodex*-positive group and the *Demodex*-negative group in symptom, lid margin abnormalities, and MG dropout. However, previous studies verified that the *Demodex*-positive group was significantly higher than the negative group in redness score, and OSDI, and was significantly lower in FBUT. They speculated that *Demodex* infection may be an important factor to cause or aggravate the damage to the ocular surface and MGs in MGD patients [10,15]. Zhang et al. [1] have reported that the ocular *Demodex*-positive group showed significantly increased scores of OSDI, lid margin abnormality, and corneal fluorescein staining compared to the ocular *Demodex*-free group. Our findings are not entirely consistent with their studies. We speculated that the probable cause was that the subjects were in the same degree of meibomian gland dropouts in the *Demodex*-positive group and *Demodex*-negative group in our study. At the same time, we found that MG dropout is more uneven in patients with *Demodex* mite infestation. However, there were no parameters to fully evaluate uneven MG morphological changes.

Therefore, we introduced the index of UAS based on MG dropout grade in a different part of meibography. In our study, the meibograde had a statistically significant difference between the normal control group and the *Demodex*-negative MGD group, but the UAS had no statistically significant difference. It can indicate that the atrophy of the *Demodex*-negative MGD group is more serious than that of the normal control group, however, their atrophy morphology is similar, and the atrophy is even. In addition, there was a statistically significant difference in meibograde and UAS between the normal control group and the *Demodex*-positive MGD group, which can illustrate that the atrophy is more serious in the *Demodex*-positive MGD group than in the normal control group and the atrophy is more uneven. There was no statistically significant difference in meibograde between the *Demodex*-positive group and MGD group. However, the *Demodex*-positive group was significantly larger than the *Demodex*-negative group in UAS of upper eyelid or lower eyelid or both eyelids adjusted for age, suggesting that there was a significant unevenness in the atrophy of MGs in the patients infected by *Demodex*. This finding left us one hint that meibograde could not distinguish *Demodex* infestation patients from MGD patients, but UAS could. Therefore, UAS is a characteristic morphological index to identify the *Demodex* mites infestation. We hypothesized that uneven atrophy might associate with the distribution of mites. The number of mites was different in different MG regions, which might lead to different MG atrophy. At present, we usually suspect *Demodex* infection mainly based on clinical symptoms and abnormal eyelashes with cylindrical dandruff. Then, *Demodex* infection was diagnosed by positive results of eyelashes light microscopic examination or confocal microscopy. The UAS of MGs provides us with a new auxiliary diagnostic indicator for *Demodex* infection. Meanwhile, meibography is a rapid and non-invasive examination commonly used to examine dry eye. Therefore, the establishment of UAS can help to diagnose *Demodex* infection and reduce missed diagnoses.

*Demodex* mites could cause microstructural changes in the MGs, with more severe structural damage in MGD [29,30]. Lee et al. [10] reported that *Demodex* had been associated with dry eye but not necessarily with aqueous tear deficiency, and patients with mite infestation might have normal Schirmer test scores. According to the correlation analysis, we found the UAS index had significant positive correlations with OSDI, lid margin score, and meiboscore, and a significant negative correlation with TBUT (*p* < 0.05). However, there were no significant correlations between UAS and CFS, and TMH. These results confirmed that mites infection might interfere with lipid layer of tear film, and less affect the water layer of tear film.

The present study has some limitations. The index of UAS has a good evaluation effect for mild and moderate MGs dropout, but for severe MGs atrophy, as the glands in each partition are close to 0 and meibograde is close to grade 3, UAS will be close to 0 and lose diagnostic efficacy. The index of UAS is a rough semi-quantitative index, and a more accurate quantitative index is needed in further studies. In addition, the tarsus area was divided into five equal parts in our study. The more equally divided areas, the more accurate the result. Ideally, it is the most accurate to calculate UAS by the amount of atrophy in each gland region. However, manual work may be unbearable due to a large number of annotations and calculations. The aid of a computer is required, and subsequent research can rely on artificial intelligence in order to reduce workload, improve efficiency, and enhance convenience for clinical applications.

## 5. Conclusions

In summary, we propose a novel index of UAS, which is used to evaluate uneven atrophy of MGs and as a morphological index of *Demodex* infestation. Furthermore, our findings suggest that *Demodex* infestation can cause uneven MG atrophy.

## Figures and Tables

**Table 1:** Patient basic characteristics and the clinical parameters of three groups.

	Normal Control Group	*Demodex*-Negative MGD Group	*Demodex*-Positive MGD Group	*p*-Value 1	*p*-Value 2	*p*-Value 3
Age (years), Median (IQR)	25.0 (22.0, 32.0)	40.0 (30.0, 48.0)	38.5 (28.0, 55.5)	<0.001	<0.001	1.000
Eyes, n	43	49	66	-	-	-
Gender, (n, male/female)	18/25	19/30	20/46	-	-	-
OSDI, Median (IQR)	2.08 (1.19, 8.33)	37.50 (25.00, 54.55)	26.05 (13.02, 41.67)	<0.001	<0.001	0.120
TMH (mm), Median (IQR)	0.20 (0.18, 0.24)	0.22 (0.18, 0.26)	0.20 (0.18, 0.24)	0.466	0.799	0.569
TBUT (seconds), Median (IQR)	8.00 (5.00, 15.00)	2.83 (1.60, 3.08)	2.00 (1.00, 3.00)	<0.001	<0.001	1.000
CFS (0–20), Median (IQR)	0.00 (0.00, 0.00)	0.00 (0.00, 2.00)	0.00 (0.00, 2.00)	0.044	0.150	0.424
Lid margin score (0–4), Median (IQR)	0.00 (0.00, 1.00)	2.00 (2.00, 2.00)	2.00 (2.00, 3.00)	<0.001	<0.001	0.442
Meibograde (0–6), Median (IQR)	1.00 (0.00, 1.00)	2.00 (1.00, 3.00)	2.00 (1.00, 3.00)	<0.001	<0.001	1.000

*p*-value 1: Normal control group vs. *Demodex*-negative MGD group; *p*-value 2: Normal control group vs. *Demodex*-positive MGD group; *p*-value 3: *Demodex*-negative MGD group vs. *Demodex*-positive MGD group.

**Table 2:** Uneven atrophy score and meibograde in three groups.

	Normal Control Group	*Demodex* Negative Group	*Demodex* Positive Group	*p*-Value 1	*p*-Value 2	*p*-Value 3	* *p*-Value 1	* *p*-Value 2	* *p*-Value 3
UAS of upper eyelid	1.00 (0.00, 1.00)	2.00 (1.00, 3.00)	3.00 (2.00, 4.00)	0.021	<0.001	0.037	0.100	<0.001	0.042
UAS of lower eyelid	2.00 (1.00, 3.00)	2.00 (1.00, 3.00)	4.00 (3.00, 6.00)	0.707	0.085	0.045	0.502	0.213	0.020
UAS of both eyelids	3.00 (2.00, 4.00)	3.00 (2.00, 3.00)	5.00 (4.00, 6.00)	0.301	0.006	0.014	0.531	0.001	0.003
Meibograde of upper eyelid	0.00 (0.00, 0.00)	1.00 (0.00, 1.00)	1.00 (0.00, 1.00)	<0.001	<0.001	1.000	<0.001	<0.001	0.547
Meibograde of lower eyelid	1.00 (0.00, 1.00)	1.00 (1.00, 2.00)	1.00 (1.00, 2.00)	<0.001	<0.001	1.000	<0.001	<0.001	0.677
Meibograde of both eyelids	1.00 (0.00, 1.00)	2.00 (0.00, 3.00)	2.00 (1.00, 3.00)	<0.001	<0.001	1.000	<0.001	<0.001	0.960

*p*-value 1: Normal control group vs. *Demodex*-negative MGD group; *p*-value 2: Normal control group vs. *Demodex*-positive MGD group; *p*-value 3: *Demodex*-negative MGD group vs. *Demodex*-positive MGD group. * *p* values adjusted for age by generalized estimating equation.

**Table 3:** The number (ratio) of uneven atrophy score in three groups.

	UAS	Normal Control Group (n = 43)	*Demodex* Negative Group (n = 49)	*Demodex* Positive Group (n = 66)
upper eyelid	0–3	42 (97.67%)	42 (85.71%)	49 (74.24%)
	4–6	1 (2.33%)	7 (14.29%)	17 (25.76%)
	7–9	0	0	0
	10–12	0	0	0
lower eyelid	0–3	39 (90.70%)	43 (87.76%)	55 (83.33%)
	4–6	4 (9.30%)	6 (12.24%)	11 (16.67%)
	7–9	0	0	0
	10–12	0	0	0
both eyelids	0–3	21 (48.84%)	14 (28.57%)	9 (13.64%)
	4–6	21 (48.84%)	28 (57.14%)	42 (63.64%)
	7–9	1 (2.33%)	7 (14.29%)	14 (21.21%)
	10–12	0	0	1 (1.52%)

**Table 4:** Correlation analysis between UAS and ocular surface parameters.

R-Value	OSDI	TMH	TBUT	CFS	Lid Margin Score	Meibograde
UAS of upper eyelid	0.293 ^‡^	0.037	−0.304 ^‡^	−0.063	0.298 ^‡^	0.329 ^‡^
UAS of lower eyelid	0.001	0.058	−0.080	0.030	0.175 *	0.245 *
UAS of both eyelids	0.209 *	0.064	−0.248 *	−0.008	0.287 ^‡^	0.356 ^‡^

* *p* < 0.05; ^‡^ *p* < 0.001.

## Data Availability

The data presented in this study are available on request from the corresponding author.

## References

[1] Zhang X.B., Ding Y.H., He W. (2018). The association between demodex infestation and ocular surface manifestations in meibomian gland dysfunction. Int. J. Ophthalmol..

[2] Basta-Juzbasić A., Subić J.S., Ljubojević S. (2002). Demodex folliculorum in development of dermatitis rosaceiformis steroidica and rosacea-related diseases. Clin. Dermatol..

[3] Cheng A.M.S., Sheha H., Tseng S.C.G. (2015). Recent advances on ocular Demodex infestation. Curr. Opin. Ophthalmol..

[4] Kabatas N., Dogan A.S., Kabatas E.U., Acar M., Bicer T., Gurdal C. (2017). The Effect of Demodex Infestation on Blepharitis and the Ocular Symptoms. Eye Contact Lens.

[5] Yildiz-Tas A., Arici C., Mergen B., Sahin A. (2021). In Vivo Confocal Microscopy in Blepharitis Patients with Ocular Demodex Infestation. Ocul. Immunol. Inflamm..

[6] Kheirkhah A., Casas V., Li W., Raju V.K., Tseng S.C. (2007). Corneal manifestations of ocular demodex infestation. Am. J. Ophthalmol..

[7] English F.P., Nutting W.B. (1981). Demodicosis of ophthalmic concern. Am. J. Ophthalmol..

[8] Liang L., Liu Y., Ding X., Ke H., Chen C., Tseng S.C.G. (2018). Significant correlation between meibomian gland dysfunction and keratitis in young patients with Demodex brevis infestation. Br. J. Ophthalmol..

[9] Rabensteiner D.F., Aminfar H., Boldin I., Nitsche-Resch M., Berisha B., Schwantzer G., Horwath-Winter J. (2019). Demodex Mite Infestation and its Associations with Tear Film and Ocular Surface Parameters in Patients with Ocular Discomfort. Am. J. Ophthalmol..

[10] Lee S.H., Chun Y.S., Kim J.H., Kim E.S., Kim J.C. (2010). The relationship between demodex and ocular discomfort. Investig. Ophthalmol. Vis. Sci..

[11] Cheng S.N., Jiang F.G., Chen H., Gao H., Huang Y.K. (2019). Intense Pulsed Light Therapy for Patients with Meibomian Gland Dysfunction and Ocular Demodex Infestation. Curr. Med. Sci..

[12] Gao Y.Y., Di Pascuale M.A., Li W., Liu D.T., Baradaran-Rafii A., Elizondo A., Kawakita T., Raju V.K., Tseng S.C. (2005). High prevalence of Demodex in eyelashes with cylindrical dandruff. Investig. Ophthalmol. Vis. Sci..

[13] Liang L., Ding X., Tseng S.C. (2014). High prevalence of demodex brevis infestation in chalazia. Am. J. Ophthalmol..

[14] English F.P., Cohn D., Groeneveld E.R. (1985). Demodectic mites and chalazion. Am. J. Ophthalmol..

[15] Pan S., Chen Y. (2021). A clinical study on the correlation between demodex infestation and ocular surface changes in patients with meibomian gland dysfunction. Indian J. Ophthalmol..

[16] Huo Y.N., Mo Y.P., Wu Y.Y., Fang F., Jin X.M. (2021). Therapeutic effect of intense pulsed light with optimal pulse technology on meibomian gland dysfunction with and without ocular Demodex infestation. Ann. Transl. Med..

[17] Zhang Z.H., Lin X.L., Yu X.X., Fu Y.N., Chen X.Y., Yang W.H., Dai Q. (2022). Meibomian Gland Density: An Effective Evaluation Index of Meibomian Gland Dysfunction Based on Deep Learning and Transfer Learning. J. Clin. Med..

[18] Lin X.L., Fu Y.N., Li L., Chen C.Q., Chen X.W., Mao Y.Y., Lian H.L., Yang W.H., Dai Q. (2020). A Novel Quantitative Index of Meibomian Gland Dysfunction, the Meibomian Gland Tortuosity. Transl. Vis. Sci. Technol..

[19] Yin Y., Gong L. (2015). Uneven Meibomian Gland Dropout Over the Tarsal Plate and its Correlation with Meibomian Gland Dysfunction. Cornea.

[20] Li Y., Lu J., Zhou Q.Z., Wang C.X., Zeng Q.Y., Chen T.H., Liu C., Kang Y.W., Li S.W. (2020). Analysis of Clinical and Regional Distribution Characteristics of Obstructive Meibomian Gland Dysfunction in China: A Multicenter Study. Curr. Eye Res..

[21] Asian Dry Eye Association China Branch (2020). Expert Consensus on diagnosis and Treatment of Demodex blepharitis in China (2018). Zhonghua Yan Ke Za Zhi.

[22] Arita R., Itoh K., Maeda S., Maeda K., Furuta A., Fukuoka S., Tomidokoro A., Amano S. (2009). Proposed diagnostic criteria for obstructive meibomian gland dysfunction. Ophthalmology.

[23] Adil M.Y., Xiao J., Olafsson J., Chen X., Lagali N.S., Raeder S., Utheim O.A., Dartt D.A., Utheim T.P. (2019). Meibomian Gland Morphology Is a Sensitive Early Indicator of Meibomian Gland Dysfunction. Am. J. Ophthalmol..

[24] Georgala S., Katoulis A.C., Kylafis G.D., Koumantaki-Mathioudaki E., Georgala C., Aroni K. (2001). Increased density of Demodex folliculorum and evidence of delayed hypersensitivity reaction in subjects with papulopustular rosacea. J. Eur. Acad. Dermatol. Venereol..

[25] Forton F., Germaux M.A., Brasseur T., De Liever A., Laporte M., Mathys C., Sass U., Stene J.J., Thibaut S., Tytgat M. (2005). Demodicosis and rosacea: Epidemiology and significance in daily dermatologic practice. J. Am. Acad. Dermatol..

[26] Gao Y.Y., Di Pascuale M.A., Elizondo A., Tseng S.C. (2007). Clinical treatment of ocular demodecosis by lid scrub with tea tree oil. Cornea.

[27] Tanriverdi C., Balci O., Demirci G., Odabasi M., Ozsutcu M., Nurozler Tabakci B. (2020). Comparison of Biomicroscopy and Light Microscopy Findings in Demodex Diagnosis in Patients with Chronic Blepharitis. Eye Contact Lens.

[28] Karincaoglu Y., Bayram N., Aycan O., Esrefoglu M. (2004). The clinical importance of demodex folliculorum presenting with nonspecific facial signs and symptoms. J. Dermatol..

[29] Gao H., Chen H., Xie H.T., Xu K.K., Shi B.J., Huang Y.K. (2021). Changes in Meibum Lipid Composition with Ocular Demodex Infestation. Transl. Vis. Sci. Technol..

[30] Liang X., Li Y., Xiong K., Chen S., Li Z., Zhang Z., Xia Z., Yi G., Fu M. (2021). Demodex Infection Changes Ocular Surface Microbial Communities, in Which Meibomian Gland Dysfunction May Play a Role. Ophthalmol. Ther..

